# Advanced Autumn Migration of Sparrowhawk Has Increased the Predation Risk of Long-Distance Migrants in Finland

**DOI:** 10.1371/journal.pone.0020001

**Published:** 2011-05-18

**Authors:** Aleksi Lehikoinen

**Affiliations:** Finnish Museum of Natural History, University of Helsinki, Helsinki, Finland; Pennsylvania State University, United States of America

## Abstract

Predation affects life history traits of nearly all organisms and the population consequences of predator avoidance are often larger than predation itself. Climate change has been shown to cause phenological changes. These changes are not necessarily similar between species and may cause mismatches between prey and predator. Eurasian sparrowhawk *Accipiter nisus*, the main predator of passerines, has advanced its autumn phenology by about ten days in 30 years due to climate change. However, we do not know if sparrowhawk migrate earlier in response to earlier migration by its prey or if earlier sparrowhawk migration results in changes to predation risk on its prey. By using the median departure date of 41 passerine species I was able to show that early migrating passerines tend to advance, and late migrating species delay their departure, but none of the species have advanced their departure times as much as the sparrowhawk. This has lead to a situation of increased predation risk on early migrating long-distance migrants (LDM) and decreased the overlap of migration season with later departing short-distance migrants (SDM). Findings highlight the growing list of problems of declining LDM populations caused by climate change. On the other hand it seems that the autumn migration may become safer for SDM whose populations are growing. Results demonstrate that passerines show very conservative response in autumn phenology to climate change, and thus phenological mismatches caused by global warming are not necessarily increasing towards the higher trophic levels.

## Introduction

Predation affects life history traits of nearly all organisms [1]. A large diversity of anti-predator mechanisms has evolved [2] leading to costly indirect effects of predation [3]. Ongoing global warming may affect the predator-prey interactions, since species show different phenological changes due to increasing temperature [4]. These phenological changes may cause mismatches between predator and prey [5], [6], [7], which may have population consequences [8]. Climate driven mismatches have been shown on all trophic levels and mistiming appears to increase at higher hierarchies of the food chain [9].

Warming spring temperatures have changed the timing of life history events of a small-sized predator, the Eurasian Sparrowhawk *Accipiter nisus*, in Finland. April temperatures have increased in the southern part of Finland by about 2.2°C during 1973–2007 [10]. Increasing temperature has advanced the early phase of arrival and breeding dates, which has lead to earlier initial and median dates of departure from Finland [10]. Since the sparrowhawk is the main predator of European passerines, a changing phenology of the predator could be caused by changes in phenology of its prey (bottom up) or alternatively, an advanced sparrowhawk phenology could cause changes in predation risk on prey species. In spring, both the sparrowhawk and many passerines have advanced their arrival [10], [11], [12]. However, the situation is not that well known for the autumn migration and the few studies dealing with the topic have shown contradictory results in passerines varying from an advancement to a delay in autumn departure dates [13], [14], [15].

For this paper I studied the following questions, i) has the phenology of 41 passerine species changed during the last 30 years and ii) has the autumn migration predation risk on sparrowhawk prey species changed due to the advanced departure of the predator. The sparrowhawk migration season is fairly long in Northern Europe starting from August and ending in early November. This broad migration season is because different age and sex classes have different departure dates although there are large overlaps between the groups. Young birds migrate first, especially in August and September, while adults migrate later after moult of flight feathers mainly in October [16], [17]. Hence, the advance of early and median phases of the departure mainly concerns young individuals [10], [16], [17].

## Results

**Figure 1:**
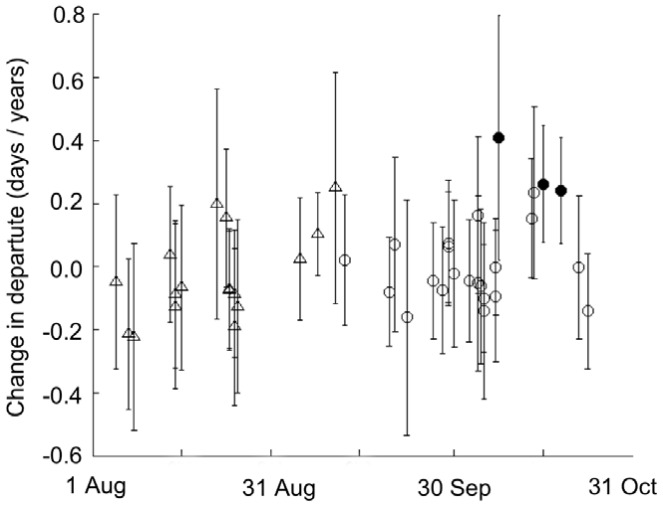
Changes in departure dates (days/year) on short- (circles) and long-distance (triangles) migrating passerines in 1979–2008. Bars represents the standard errors of the coefficients. Three species, that have significantly delayed their migration, are shown in black circles.

**Figure 2:**
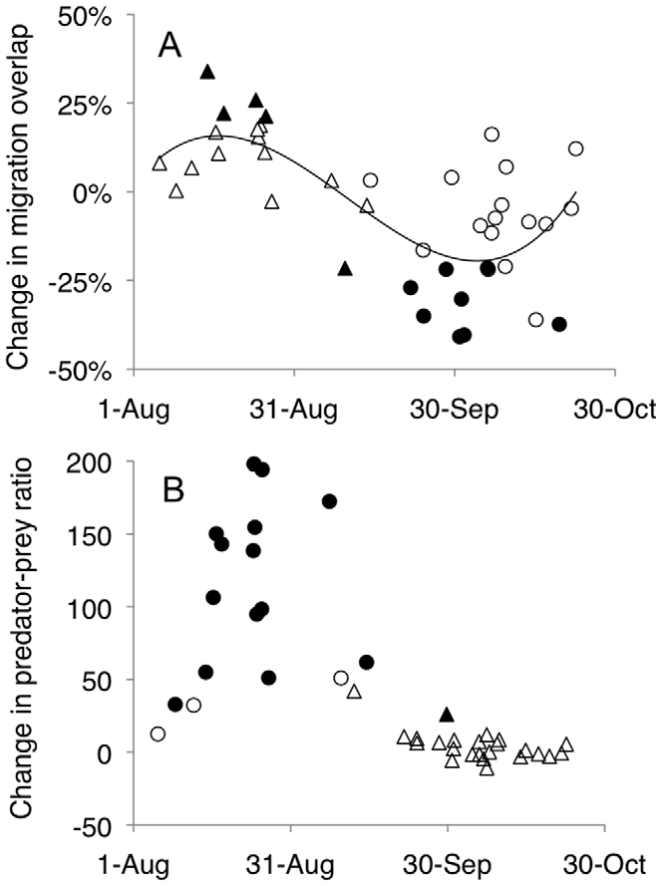
Changes in (A) overlap of migration season between sparrowhawk and short- (circles) and long-distance (triangles) migrating passerines and (B) changes in relative predation risk (predators/1000 prey individuals) in same species in 1979–2008. Filled symbols indicate significant changes of particular species. In Fig. 2A 1% change in predation pressure means 1% absolute change in the total flyway population of sparrowhawks migrating within the main migration season of the prey species (*e.g.* 100% mean in 1979 no sparrowhawks migrated within the migration season of the species, but in 2008 all sparrowhawks migrated within the same time of the species). Furthermore, the third-degree polynomial model (r^2^ = 0.51) in Fig. 1A was significantly better than the linear model (ΔAICc = 5.2, ER = 13.8) and null model (ΔAICc = 21.9, ER>10000).

Results show that as a group neither short-distance migrants (SDM; three significantly delaying species) nor long-distance migrants (LDM; no significant changes) have changed their median migration date significantly (Table S1). There was also no significant difference between these groups (SDM: mean change 0.028 days/year±0.030 SE, n = 24; LDM: −0.032±0.034 SE, n = 17; Fig. 1A). However, there was a significant trend that early migrating species tended to advance, and late migrating species tended to delay, their departure dates (r_s_ = 0.32, df = 39, P = 0.044, Fig. 1, Table S1). Nevertheless, the overlap between migration season of sparrowhawks and several passerine species has changed: a larger proportion of the sparrowhawk population nowadays migrates within the migration period of LDM, whereas the overlap of sparrowhawk and SDM migration periods has reduced (Fig. 2A, Table S1). The predation risk of LDM has also increased when risk is analysed as the daily number of sparrowhawks per prey individuals during the migration season of each passerine species even if observed con- and heterospecific prey individuals are included (Fig. 2B, Table S1). In contrast, the same analysis suggests that the predation risk on SDM has not significantly changed (Fig. 2B, Table S1).

## Discussion

Results highlight that the phenological changes in departure dates of passerines are very conservative, despite both August and September temperatures in southern part of Finland having increased 2.1–2.4°C since the mid-1970s [10]. Surprisingly, I did not find a difference between phenological changes in LDM and SDM, unlike in earlier studies [13], [15]. The mean slopes for SDM and LDM are nearly equal suggesting that the sample size of 41 species did not present insufficient power to detect a difference. Only three species, all partial SDM with potential for multiple broods per breeding season [18], showed significantly delayed departure dates. Interestingly, none of the passerine species advanced their departure dates as much as the sparrowhawk, and in general the rate of advance was ten times higher in sparrowhawk than in LDM (Fig. 1) [10]. This has lead to a situation where the predation risk facing several passerine species has changed with time. Some early departing warbler species can still migrate mainly before the sparrowhawks (extreme left part in Fig. 2A), but most of the LDM that depart during August need to cope with increased sparrowhawk densities during their migration (peak phase of the polynomial fit in Fig. 2A). SDM in contrast experience decreasing migration overlap with sparrowhawk while some species are still migrate late enough to avoid most sparrowhawk (extreme right observations in Fig. 2A). Advancing autumn migration can be beneficial for sparrowhawk, permitting increased overlap with prey, both migrating LDM and local SDM, which are preparing for their coming migration. These results therefore suggest that phenological mismatches due to climate change are not necessary becoming higher with increasing trophic level [8].

LDM are displaying more general declines in population densities compared to SDM [19], [20], [21], whose populations have typically remained stable or even increased [20], [22]. The predator/prey ratio did not show decreasing trend in SDM even though their migration season overlaps less with sparrowhawks. This is likely due to increasing numbers of migrating sparrowhawk [23], perhaps caused by improved sparrowhawk breeding success during the study period, for example, due to a decrease of environmental toxins [10], [24], [25]. Therefore the actual number of sparrowhawks migrating within migration seasons of SDM has not changed even though the overlap of migration periods has reduced. Nevertheless, if sparrowhawk continue to advance their autumn migration it could be beneficial for SDM to delay autumn departure. Maintaining the current trends would lead to decreased future predation risk for SDM during autumn migration. Predation pressure during migration for LDMs will however increase.

One may wonder whether changes in predation risk could have any population effects. In the UK, sparrowhawk populations have recovered from the collapse caused by pesticides, and this population increase could have caused significant population declines of resident house *Passer domesticus* and tree sparrows *P. montanus* [26], [27], but in other prey species there is no such evidence [24], [26], [27]. Nevertheless, higher predation risk not only causes increased mortality, but at the population level, the negative non-lethal effects can be even larger than direct predation effects, through costly investment in anti-predator behaviours [3], [28]. During breeding passerines are known to avoid breeding close to sparrowhawk nests [29], [30]. Indeed, individuals that breed in close proximity to sparrowhawk nests suffer costs in terms of decreased body condition, higher stress protein induction and immunoglobulin levels [31] and their overall reproductive output is lowered [30], [32]. Since predation risk can create areas that are avoided by prey [30], [33], [34], affect the physiological condition of prey species [31], [35] and lead to later breeding [33], increased predation may reduce feeding efficiency along the migration route and lead to longer staging periods and poorer body condition. Migration is already demanding and risky [36], and it is becoming increasingly risky as predator densities increase [29]. Increasing predation risk combined with worsening stopover conditions [21] could entail crucial negative impacts on survival.

Sparrowhawk departure dates in southern France have also advanced at exactly the same rate as in Finland [10], [37], which emphasizes that changes in predation risk in migratory passerines are likely large-scale phenomena in Europe. The decline of LDM has been argued to be related to increased mismatches during arrival and breeding leading to hampered breeding success [7], [8], [38], [39] whereas declining wintering or stopover conditions are causing lower survival of fully grown birds [19], [21]. The results of this paper show that LDM are facing increasing problems also during autumn migration. Therefore, the findings add to the growing list of problems encountered by LDM because of climate change. This emphasizes the need for conservation actions focussed on this group.

## Material and Methods

The phenology of migratory bird species were monitored at the Hanko Bird Observatory, SW Finland 1979–2008 using standardized migration counts (including four hour standardized migration observation from the sunrise and standardized counts of staging birds) and trapping data (including standardized mist-netting sites, where vegetation cover is kept constant) from 25 July to 5 November [40], [41]. The combined data of all observation activities including the number of trapped birds resulted in a daily bird count. Observation activity covered 97% (81–100%) of the observation dates annually, and there was no trend in observation phenology (r_s_ = −0.15, P = 0.42, r_s_ = −0.06, P = 0.74, r_s_ = 0.05, P = 0.79, for 5%, 50% and 95%, respectively). All the observation and trapping at the observatory have been performed under study permit of Finnish environmental authorities (Environmental Centres, nowadays Centre for Economic Development, Transport and the Enviroment).

For the analyses I chose 41 common small-sized passerine species, which are suitable prey for sparrowhawk, and that exhibit their main migration timing within the main observation period [40]. Selected species were also common enough so that at least 20 individuals were observed during each migration period [40], [41]. I divided the species into LDM (wintering areas mainly south from Sahara desert) and SDM (wintering areas in Europe or Mediterranean region [42]. I calculated annual median dates for each species based on daily observation values. I use median dates since this is more conservative than a single peak date, which could be caused by exceptional weather conditions.

The potential changes in the predation risk can be analysed in two ways: i) has the overlap between migration seasons changed and ii) has the ratio of predator to prey changed, based on actual numbers when taking con- and heterospecifics into account. I calculated the median migration date and annual main migration periods (dates between 5% and 95% percentiles of migrating numbers) [40], [41] for the 41 passerine species. I used linear regression for estimating the rate of change in the annual median departure date over the study period. To evaluate the potential changes in overlap between migration seasons, I calculated the proportion of sparrowhawks (both young and adult birds included) migrating within the annual main migration period of each passerine species, with the time period between 5 and 95 percentiles of the migration. This annual proportion was regressed with year to find out the possible change in predation risk. Furthermore, to estimate potential changes in the ratio of predators and prey, I divided the daily number of sparrowhawk with the number of prey (sum of all observed individuals of these 41 species). These daily predation risk values (sparrowhawk/prey) were multiplied with the species-specific daily proportions of migration phenology for every autumn season. The results are shown as species-specific changes on the number of sparrowhawks/1000 prey individuals during the 30 years study period. This ratio cannot been taken as an accurate ratio since larger sparrowhawk are more conspicuous than smaller passerines. Nevertheless, since data is collected similarly every year annual ratios can be compared.

## Supporting Information

Table S1 Changes in departure dates (days/year ± SE), median migration date and change in migration time overlap with sparrowhawk (change in migration overlap/year ± SE ) and relative predation risk ratio (change in number of predators per 1000 prey individuals/year ± SE) of 17 long-distance (L) and 24 short-distance migrants (S) during 1979–2008 in South Finland. Bolded coefficient in departure dates, migration overlap and predator-prey ratio are bolded.
(DOC)
