# Supplementary material for: Advanced Autumn Migration of Sparrowhawk Has Increased the Predation Risk of Long-Distance Migrants in Finland
Source: PLoS One. 2011 May 18;6(5):e20001. doi: 10.1371/journal.pone.0020001 (PMC3097240; doi:10.1371/journal.pone.0020001)
Supplement: Table S1 — Changes in departure dates (days/year ± SE), median migration date and change in migration time overlap with sparrowhawk (change in migration overlap/year ± SE ) and relative predation risk ratio (change in number of predators per 1000 prey individuals/year ± SE) of 17 long-distance (L) and 24 short-distance migrants (S) during 1979–2008 in South Finland. Bolded coefficient in departure dates, migration overlap and predator-prey ratio are bolded. (DOC) [file pone.0020001.s001.doc]

Table S1. Changes in departure dates (days / year ± SE), median migration date and change in migration time overlap with sparrowhawk (change in migration overlap / year ± SE ) and relative predation risk ratio (change in number of predators per 1000 prey individuals / year ± SE) of 17 long-distance (L) and 24 short-distance migrants (S) during 1979–2008 in South Finland. Bolded coefficient in departure dates, migration overlap and predator-prey ratio are bolded.

| Species and migration strategy | Change in departure dates | Median | Change in migration overlap | Change in predator/prey -ratio |
| --- | --- | --- | --- | --- |
| Tree pipit *Anthus trivialis*, L | -0.126 ± 0.131 | 26-Aug | -0.0009 ± 0.0031 | **1.70 ± 0.61** |
| Red-throated pipit *Anthus cervinus*, L | 0.104 ± 0.066 | 9-Sep | **-0.0072** ± 0.0028 | 1.40 ± 1.25 |
| Yellow wagtai *Motacilla flava*, L | -0.076 ± 0.095 | 25-Aug | 0.0052 ± 0.0029 | **5.15 ± 1.35** |
| Redstart *Phoenicurus phoenicurus*, L | 0.025 ± 0.097 | 6-Sep | 0.0011 ± 0.0030 | **5.75 ± 1.56** |
| Whinchat *Saxicola rubetra*, L | -0.087 ± 0.101 | 26-Aug | 0.0062 ± 0.0033 | **4.62 ± 1.62** |
| Northern wheatear *Oenanthe oenanthe*, L | 0.199 ± 0.183 | 23-Aug | **0.0086** ± 0.0031 | **3.16 ± 0.85** |
| Icterine warbler *Hippolais icterina*, L | -0.048 ± 0.139 | 6-Aug | 0.0027 ± 0.0015 | 0.42 ± 0.21 |
| Blackcap *Sylvia atricapilla*, L | 0.250 ± 0.183 | 12-Sep | 0.0011 ± 0.0017 | 2.06 ± 0.99 |
| Garden warbler *Sylvia borin*, L | -0.070 ± 0.095 | 25-Aug | **0.0071** ± 0.0033 | **6.47 ± 1.63** |
| Lesser whitethroat *Sylvia curruca*, L | -0.089 ± 0.117 | 16-Aug | 0.0056 ± 0.0037 | **3.55 ± 0.98** |
| Whitethroat *Sylvia communis*, L | -0.214 ± 0.119 | 8-Aug | 0.0001 ± 0.0035 | **1.09 ± 0.31** |
| Wood warbler *Phylloscopus sibilaxtris*, L | -0.126 ± 0.138 | 16-Aug | 0.0036 ± 0.0037 | **5.01 ± 1.69** |
| Willow warbler *Phylloscopus trochilus*, L | 0.155 ± 0.110 | 24-Aug | 0.0059 ± 0.0030 | **6.60 ± 1.73** |
| Spotted flycatcher *Muscicapa striata*, L | -0.191 ± 0.124 | 26-Aug | 0.0037 ± 0.0030 | **3.28 ± 1.02** |
| Pied flycatcher *Ficedula hypoleuca*, L | -0.065 ± 0.131 | 17-Aug | **0.0074** ± 0.0030 | **4.77 ± 1.32** |
| Red-backed shrike *Lanius collurio*, L | -0.222 ± 0.148 | 9-Aug | 0.0022 ± 0.0040 | 1.08 ± 0.77 |
| Scarlet rosefinch *Carpodacus erytrinus*, L | 0.038 ± 0.108 | 15-Aug | **0.0113** ± 0.0025 | **1.83 ± 0.75** |
| Skylark *Alauda arvensis*, S | 0.164 ± 0.125 | 6-Oct | -0.0073 ± 0.0039 | -0.05 ± 0.28 |
| Meadow pipit *Anthus pratensis*, S | -0.046 ± 0.092 | 28-Sep | **-0.0073** ± 0.0031 | 0.22 ± 0.37 |
| White wagtail *Motacilla alba*, S | 0.021 ± 0.103 | 13-Sep | -0.0013 ± 0.0017 | 1.40 ± 0.69 |
| Wren *Troglodytes troglodytes*, S | -0.101 ± 0.086 | 7-Oct | 0.0054 ± 0.0038 | 0.40 ± 0.27 |
| Dunnock *Prunella modularis*, S | -0.080 ± 0.087 | 21-Sep | **-0.0090** ± 0.0029 | 0.36 ± 0.54 |
| Eurasian robin *Erithacus rubecula*, S | -0.075 ± 0.101 | 30-Sep | 0.0013 ± 0.0037 | **0.87 ± 0.37** |
| Song thrush *Turdus philomelos*, S | -0.022 ± 0.117 | 2-Oct | **-0.0135** ± 0.0039 | 0.28 ± 0.37 |
| Redwing *Turdus iliacus*, S | 0.235 ± 0.136 | 15-Oct | -0.0120 ± 0.0063 | 0.04 ± 0.17 |
| Mistle thrush *Turdus viscivorus*, S | -0.053 ± 0.139 | 6-Oct | -0.0039 ± 0.0032 | -0.36 ± 0.37 |
| Chiffchaff *Phylloscopus collybita*, S | 0.063 ± 0.088 | 1-Oct | **-0.0136** ± 0.0040 | -0.19 ± 0.51 |
| Goldcrest *Regulus regulus*, S | -0.142 ± 0.092 | 7-Oct | **-0.0072** ± 0.0030 | 0.24 ± 0.28 |
| Blue tit *Parus caeruleus*, S | -0.063 ± 0.122 | 6-Oct | -0.0025 ± 0.0020 | -0.15 ± 0.14 |
| Great tit *Parus major*, S | -0.093 ± 0.104 | 9-Oct | -0.0012 ± 0.0021 | 0.01 ± 0.15 |
| Treecreeper *Certhia familiaris*, S | **0.410** ± 0.194 | 9-Oct | 0.0023 ± 0.0040 | 0.29 ± 0.14 |
| Chaffinch *Fringilla coelebs*, S | 0.070 ± 0.138 | 22-Sep | **-0.0117** ± 0.0036 | 0.31 ± 0.26 |
| Brambling *Fringilla montifringilla*, S | -0.044 ± 0.097 | 4-Oct | -0.0032 ± 0.0029 | -0.05 ± 0.14 |
| Greenfinch *Carduelis chloris*, S | **0.262** ± 0.093 | 17-Oct | -0.0030 ± 0.0020 | -0.03 ± 0.13 |
| Goldfinch *Carduelis carduelis*, S | 0.154 ± 0.095 | 15-Oct | -0.0028 ± 0.0036 | -0.11 ± 0.19 |
| Siskin *Carduelis spinus*, S | -0.161 ± 0.187 | 24-Sep | -0.0055 ± 0.0037 | 0.21 ± 0.30 |
| Linnet *Carduelis cannabina*, S | -0.001 ± 0.077 | 9-Oct | -0.0070 ± 0.0056 | 0.20 ± 0.16 |
| Redpoll *Carduelis flammea*, S | -0.142 ± 0.140 | 24-Oct | 0.0040 ± 0.0051 | 0.18 ± 0.17 |
| Northern bullfinch *Pyrrhula pyrrhula*, S | -0.002 ± 0.114 | 23-Oct | -0.0016 ± 0.0022 | -0.02 ± 0.14 |
| Yellowhammer *Emberiza citrinella*, S | **0.242** ± 0.084 | 20-Oct | **-0.0125** ± 0.0037 | -0.09 ± 0.14 |
| Reed bunting *Emberiza schoeniclus*, S | 0.075 ± 0.100 | 1-Oct | **-0.0101** ± 0.0048 | 0.08 ± 0.26 |
